# Mapping a *Toxoplasma gondii* interactome by crosslinking mass spectrometry and machine learning

**DOI:** 10.1128/mbio.02159-25

**Published:** 2025-08-28

**Authors:** Tadakimi Tomita, Elizabeth Weyer, Rebekah B. Guevara, Simone Sidoli, Jennifer T. Aguilan, Louis M. Weiss

**Affiliations:** 1 Department of Pathology, Albert Einstein College of Medicine 2006, New York, New York, USA; 2 Department of Biochemistry, Albert Einstein College of Medicine 2006, New York, New York, USA; 3 Department of Genetics, Albert Einstein College of Medicine 2006, New York, New York, USA; 4 Department of Medicine, Albert Einstein College of Medicine 2006, New York, New York, USA; Tsinghua University, Beijing, China

**Keywords:** *Toxoplasma gondii*, proteomics, protein crosslinking agents, machine learning, GRA, ribosome, protein-protein interactions, XL-MS, mass spectrometry, interactome

## Abstract

*Toxoplasma gondii*, a widespread human parasite, persists in hosts through complex molecular interactions. Protein-protein interactions (PPIs) underpin essential biological processes, including parasite-host interactions and cellular invasion. Herein, we utilized advanced crosslinking mass spectrometry (XL-MS) techniques to map a *T. gondii* tachyzoite cytosolic extract interactome. By integrating MS-cleavable and non-cleavable analysis, we identified a total of 196 unique PPIs at medium confidence (false discovery rate [FDR] < 5%) and 171 at high confidence (FDR < 1%), revealing both known and novel interactions within critical cellular complexes such as the ribosome, proteasome, and dense granule proteins. Structural validation confirmed spatial proximity of crosslinked residues, while comparative analyzes against existing data sets (hyperLOPIT, ToxoNET, and STRING) corroborated the biological relevance of identified interactions. Furthermore, we introduced a machine learning approach leveraging biological annotations and experimental data to significantly enhance the detection and validation of PPIs. Our findings not only provide a refined view of *T. gondii*’s molecular architecture but also highlight the utility of XL-MS coupled with computational tools in dissecting complex parasite proteomes. The XL-MS interactome map provides a new valuable resource for understanding parasite biology and developing targeted therapeutic strategies.

**IMPORTANCE:**

Our work presents a novel application of crosslinking mass spectrometry (XL-MS) integrated with machine learning to systematically characterize the cytosolic protein-protein interactions in *Toxoplasma gondii*—a pathogen of significant clinical and epidemiological interest. This study addresses an important gap in microbial proteomics by leveraging advanced XL-MS techniques to capture transient and novel interactions, which are often challenging to detect with conventional methods. By combining both MS-cleavable and non-cleavable strategies with a robust machine learning approach, we were able to significantly enhance the identification of genuine protein interactions. The methodology described not only improves the depth and accuracy of interactome analysis but also offers a framework that can be applied to other complex microbial systems. We believe that the insights gained from our study will be of great interest to the microbiology community, particularly researchers focusing on host-pathogen interactions and the molecular mechanisms underlying parasitic infections.

## INTRODUCTION

*Toxoplasma gondii* is a prevalent human parasite that is estimated to chronically infect a quarter of the human population (1). HIV-related cerebral toxoplasmosis remains a significant problem in people with HIV infection with CD4^+^ counts under 200 cells/mm^3^ (2). While it is the most common cause of inflammatory mass lesions in the central nervous system in these patients, the availability of combination antiretroviral therapy has significantly reduced the incidence of cerebral toxoplasmosis in people living with HIV/AIDS (3). Despite the lower incidence rates, it remains an important problem, and research is needed to develop new approaches to ameliorate the pathogenesis of this central nervous system illness.

Protein-protein interactions (PPIs) are vital for the understanding of the biological process in a cell. These interactions allow proteins to bind together, creating structures and forming functional biochemical machines, which regulate protein function. Such processes are instrumental in orchestrating cellular activities, including replication, movement, and specifically in the case of *T. gondii*, the infection of host cells. Therefore, cataloging proteome-wide PPIs extends beyond mere stamp collecting; it is a critical step in unraveling the intricate complexity of biological life.

PPIs are traditionally studied using methods such as affinity purification, co-immunoprecipitation, BioID-based proximity labeling, and yeast-two-hybrid methods. Affinity purification-mass spectrometry (AP-MS) isolates specific proteins from complex mixtures via tags or antibodies, followed by mass spectrometric identification. While precise, AP-MS struggles to capture weak or transient PPIs and requires genetic modifications for affinity tags and specific antibodies, making proteome-level studies challenging. Biotin proximity labeling (BioID) employs a biotin ligase fused to a target protein to covalently attach biotin to nearby proteins that may interact with the target. These biotinylated proteins are subsequently isolated via streptavidin affinity purification. Although this method is effective in detecting weak and transient interactions, it does not confirm direct physical contact and still relies on genetic tagging, which may restrict its broader applicability. Emerging techniques like photoactivable unnatural amino acid crosslinking more directly explore interactions, but also demand genetic manipulation of each protein (4). Conversely, approaches such as hyperLOPIT (5) and co-elution (6) are used for large-scale mapping of subcellular locations and protein complexes in parasites, achieving proteome-wide insights, but with lower resolution concerning specific organelles and complexes. Crosslinking mass spectrometry (XL-MS) has recently evolved into a powerful tool for studying PPIs, due to advancements in peptide mass spectrometry and computational methods. The technique works by forming covalent bonds that effectively “freeze” the proteins in their native state. A chemical crosslinker is used to impose spatial constraints that mirror the in-solution conformations of intact proteins.

A new generation of enrichable MS-cleavable crosslinkers, such as DSBSO (7), has greatly improved XL-MS. Click reactive groups have been incorporated to enrich crosslinked peptides from unmodified peptides successfully used for proteome-level studies. These include MS non-cleavable but more hydrolysis-resistant *N*-succinimidyl carbamate crosslinker NNP9 (8) and the MS-cleavable carboxyl-selective crosslinker BAP (9). Click enrichment of digested peptides rather than proteins increases the recovery of the labeled proteins (10). Bypassing biotin-streptavidin and Cu(I)-catalyzed azide-alkyne cycloaddition (CuAAC), through the simple use of strain-promoted azide-alkyne cycloaddition (SPAAC) by Dibenzocyclooctyne (DBCO) coupled beads, increases the enrichment efficiency (11). In addition, enrichment of larger peptides by size exclusion columns increases recovered crosslinks and reduces monolinks (12, 13). Use of high-pH reverse-phase fractionation further reduces the complexity of samples and expands coverage (13). On the instrument side, the use of high-field asymmetric waveform ion mobility spectrometry (FAIMS) improves depth and sensitivity by enriching highly charged ions (12, 14). Moreover, to further optimize peptide identification, stepped higher-energy collisional dissociation (HCD) fragmentation can be implemented (15).

Herein, we demonstrate that the XL-MS protocol we developed generated crosslinking data that is consistent with known PPIs and experimentally derived structures. Additionally, we developed and applied a machine learning approach to increase the number of PPIs identified in our crosslinking data set. Finally, we demonstrate the utility of XL-MS data in predicting protein structure, both of individual proteins and of protein complexes.

## MATERIALS AND METHODS

### Chemicals

Chemicals used in these experiments were purchased from commercial suppliers, for example, Azide-A-DSBSO (Millipore Sigma: 909629), DBCO agarose beads (Vector Laboratories), Trypsin Gold 100 µg (Promega), Lys-C (MedChemExpress), Protease inhibitor cocktail cOmplete EDTA free (Roche), and high pH reversed-phase peptide fractionation kit (Pierce). We observed significant variations in the quality of DSBSO across different lots from Millipore Sigma. To ensure efficient crosslinking and maintain consistency in experimental outcomes, we recommend conducting a preliminary crosslinking assay with each new lot prior to its use in large-scale experiments and/or validation of lots of DSBSO by ^1^H-NMR. The resulting spectrum should display a strong peak at 2.82 ppm (NHS ester) and show no peak at 2.60 ppm (indicative of hydrolysis).

### Cell culture

An ME49 strain of *T. gondii* with deletions of the KU80 gene (TGME49_312510) and hypoxanthine-xanthine-guanine phosphoribosyl transferase (TGME49_200320) (16) was cultured in human foreskin fibroblasts (HFFs; ATCC: CRL-1634; Hs27) in Dulbecco’s modified Eagle medium supplemented with penicillin-streptomycin at 37°C 5% CO_2_. The experiment was performed as duplicates. Eight 150 mm plates of HFF per sample were infected with parasites and cultured for 3 days. Parasites were lysed out of host cells by passing through a 27G needle three times and a 5 µm membrane filter once. Parasites were then washed three times with PBS, followed by hypotonic lysis that was performed as described previously (17). Briefly, the cell pellet was resuspended with 100 µL of 10 mM HEPES pH 7.5 with cOmplete protease inhibitor cocktail EDTA free and incubated on ice for 10 min. The lysate was spun at 16,000 × *g*, and the supernatant was harvested. Hypotonic extraction was repeated a total of five times. The soluble lysates were combined and then 10 x PBS was added to the lysate at 1 x final concentration.

### Crosslinking and digestion

The crosslinking protocol was performed following a previously published method (17) with the following modifications. Lysates were adjusted to 1 µg/µL with 1× PBS. The crosslinker Azide-A-DSBSO was added to the lysate at a concentration of 5 mM gradually to prevent precipitation of the crosslinker. This was followed by incubation for 2 h at room temperature while the tube rotated at 10 rpm. Careful optimization of incubation time (30 min to 2 h), lysate concentration (1–10 µg/µL), and crosslinker concentration (1–5 mM) is required to strike an optimal balance between maximal mass spectrometric hits and specificity. Crosslinking was terminated by adding Tris pH 8 at 50 mM and incubation for 5 min at room temperature. Digestion was performed using the filter-aided sample preparation method (18, 19) as follows: the samples were washed with 8 M urea in 25 mM ammonium bicarbonate three times using a 3 kDa nominal cutoff Amicon Ultra filter device (Millipore Sigma). The proteins were reduced with Tris (2-carboxyethyl) phosphine at 2 mM for 30 min and alkylated with iodoacetamide at 20 mM for 30 min in the dark. The samples were washed once with 8 M urea in 25 mM ammonium bicarbonate and digested in 6.4 M urea in 25 mM ammonium bicarbonate with 1:100 (wt/wt, protease:protein) LysC for 4 h at 37°C. Subsequently, the sample was diluted with 25 mM ammonium bicarbonate to 1 M urea and incubated overnight at 37°C with Trypsin Gold at 1:50 (wt/wt). After the digestion, acetonitrile was added to a final concentration of 10% and incubated with 40 µL of DBCO agarose beads per mg of initial protein at 4°C overnight. Due to their inherently hydrophobic nature, crosslinked peptides require the inclusion of acetonitrile to improve their solubility throughout the processing workflow. The DBCO beads were washed three times with 1 M NaCl and 10% acetonitrile in 25 mM ammonium bicarbonate sequentially in disposable micro columns. The peptides were eluted with 10% trifluoroacetic acid (TFA) by rotating the tube at room temperature for 30 min at 5 rpm. The beads were eluted two times, and the eluates were neutralized with ammonium bicarbonate to yield a final concentration of 100 mM ammonium bicarbonate in the tube. The beads were then eluted with 50% acetonitrile in 25 mM ammonium bicarbonate two times. All eluates were then combined and vacuum dried. The peptides were fractionated with high pH reversed-phase peptide fractionation kit following the manufacturer’s protocol (Pierce). The elution was performed with 5%, 10%, 15%, 20%, 25%, 30%, 35%, and 50% acetonitrile sequentially, and two fractions were combined (5 + 25%, 10 + 30%, 15 + 35%, and 20 + 50%) to make four fractions per sample.

### Crosslinked peptide enrichment by size exclusion chromatography (SEC)

An additional sample was enriched using SEC rather than high-pH fractionation. Following click chemistry-based purification, the dried peptides were dissolved in 30% acetonitrile (ACN) with 0.1% TFA to achieve a concentration of 20 µg/µL. Two hundred micrograms of peptides were then injected into a Superdex 30 Increase 3.2/300 column (Cytiva) and eluted isocratically with the same buffer. Fractions of 50 µL were collected, dried, and subsequently analyzed by mass spectrometry. The result of this fractionation is provided in Fig. S1. In summary, the fraction from 1.18 to 1.23 mL exhibited the highest ratio of crosslinked to non-crosslinked species, while the fraction from 1.23 to 1.28 mL contained the greatest number of crosslinks.

### Mass spectrometric analysis

Samples were resuspended in 0.1% TFA and loaded onto a Dionex RSLC Ultimate 3000 (Thermo Scientific), coupled online with an Orbitrap Exploris 480 (Thermo Scientific) with FAIMS Pro attached. Chromatographic separation was performed with a two-column system, consisting of a C-18 trap cartridge (300 µm ID, 5 mm length) and a picofrit analytical column (75 µm ID, 25 cm length) packed in-house with reversed-phase Repro-Sil Pur C18-AQ 3 µm resin. Peptides were separated using a 180-min gradient from 4% to 30% buffer B (buffer A: 0.1% formic acid, buffer B: 80% acetonitrile + 0.1% formic acid) at a flow rate of 300 nL/min. The mass spectrometer was set to acquire spectra in a data-dependent acquisition mode. Briefly, the full MS scan was set to 375–1,600 *m/z* with a resolution of 120,000 (at 200 *m/z*). MS/MS was performed at 60,000 resolution and AGC target of 1 × 10e4 and stepped HCD normalized collision energy of 19%, 25%, and 32% (11, 15, 20) with FAIMS compensation voltages of −50, −60, and −75 (14).

The data were analyzed with Proteome Discoverer 2.5 with IMP-MS2 spectrum Processor for deisotoping and MS Amanda 2.0 with the following parameters: maximum missed cleavage 4, MS1 tolerance 10 ppm, MS2 tolerance 20 ppm, static modification: carbamidomethyl 57.021 Da (C), and dynamic modifications: oxidation 15.995 Da (M). Spectrum searches were performed on ToxoDB (toxodb.org) release 67 (21). Data sets were analyzed with MS Annika 2.0 (22) as MS-cleavable crosslinkers with the following parameters: DSBSO 308.039 Da (K), alkene 54.0156 Da, and thiol 236.0177 Da, additional crosslink doublet 200.0177 on K, S, T, and Y residues. Additionally, the data were analyzed with pLink 3 (23) as non-cleavable crosslinker with the following parameters: DSBSO 308.039 Da (linker mass) and 326.049 Da (mono mass).

### Data processing and machine learning

The data generated by Annika 2.0 and pLink 3 were processed using R (24) script with tidyverse (25) packages. Annika 2.0 reports crosslinking results as peptide pairs; therefore, false discovery rates (FDRs) were recalculated using a custom R script. First, peptide pairs were collapsed to residue-to-residue pairs and then aggregated into PPI sequentially. Quality scores were adjusted from lower level scores by calculating Euclidean norms, and the FDRs were recalculated at higher levels after inter/intra crosslinks were separated according to the previously described method (26). Ambiguous hits were referenced with the tachyzoite transcriptome data, retaining only the highest-expressing hits in each case for Annika 2.0 results. All ambiguous hits were removed for pLink results due to the report format. The hyperLOPIT (5), CRISPR fitness score (27), and GO term data were downloaded from ToxoDB release 67 (toxodb.org). The STRING data were downloaded from STRING version 12.0 (string-db.org), Uniprot names were converted to ToxoDB ID using UniProt ID mapping tool. Machine learning was performed on Google Colab with Python. The LightGBM binary classifier (28) was trained using high-confidence hits (FDR < 1%) as positive and low-confidence hits (PPI score < 10) as negative data. Missing values are not imputed but handled internally in LightGBM. Hyperparameter tuning was performed with Optuna (29) with early stopping to prevent overfitting. The resulting probabilities were used to define hierarchical cutoff thresholds: all high-confidence hits were retained; medium-confidence hits were retained if their probability exceeded 0.1; low-confidence hits were retained if their probability exceeded 0.8; and marginal hits were retained only if their probability exceeded 0.98. The Python and R scripts used in this study are available in the supplementary files (File S1A and B).

### Data visualization

Interactomes were visualized with Cytoscape 3 (30). Interactomes with residue-to-residue interactions were visualized using xiView (31). Structural mapping to the known structures and measurement of crosslink distance were performed with ChimeraX (32) with XMAS plugin (33). Distance of random K-K residues was measured using PyMOL and python script.

## RESULTS

### Determining the cytoplasmic crosslinking interactome

**Fig 1:**
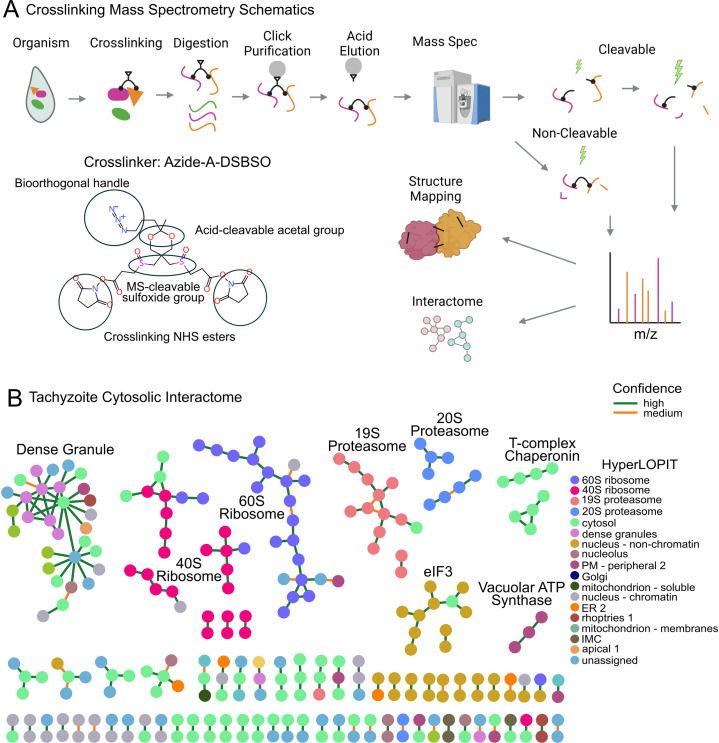
Crosslinking mass spectrometry. (**A**) Schematic illustrating the overall process of crosslinking mass spectrometry used in this study. The chemical structure shown is that of azide-a-DSBSO, the crosslinker, which contains various functional groups highlighted by circles. (**B**) Interactome networks derived from crosslinking mass spectrometry analyzes of *T. gondii* cytosolic proteins. Each node represents a protein and is color-coded according to its subcellular localization, as determined by hyperLOPIT assignments. The edges are color-coded based on the confidence of identification (high: FDR < 1%; medium: 1% < FDR < 5%).

To establish a *Toxoplasma* interactome using XL-MS, we crosslinked tachyzoite soluble cytosolic lysate. Tachyzoites were selected as the starting material since they can be produced in large quantities and readily used for crosslinking. A schematic representation of the experiment is depicted in Fig. 1A. Using the cleavable detection method, the XLMS analysis generated, at medium/high confidence (FDR < 5%/< 1%), a total of 18,692/12,993 crosslinking spectrum matches and 3,290/2,350 unique residue-residue pairs, including 3,043/2,159 intra-protein links and 247/191 inter-protein links, and 181/157 PPI from the biological triplicates (Table 1). To get the overall composition of proteins in the sample, gene ontology analysis was performed on all 157 proteins that were crosslinked at FDR < 1%. Highly significant terms include intracellular anatomical structure, ribosome, proteasome complex, cytoplasm, and translation initiation complex representing a wide variety of intracellular soluble tachyzoite lysates (File S2). To increase the number of crosslinked peptides, the same data were also searched for non-MS-cleavable crosslinks, despite using DSBSO, which is designed for the MS-cleavable protocol. This approach is based on the rationale that some crosslinked peptides may not dissociate at the crosslinker during mass spectrometry analysis, but these peptides can still fragment, allowing for their identification. Although the non-cleavable search yielded fewer results compared to the cleavable search, a substantial number of additional spectra were identified at FDRs of <1% and <5% (Table 1). This led to a 31–50% increase in residue-residue interactions (RRIs), demonstrating the benefits of this method. As a result, the identification of PPIs increased when we combined the detection methods to a total of 196 PPIs identified at an FDR < 5% and 171 PPIs at an FDR < 1%.

**TABLE 1:** The number of crosslinks identified from *T. gondii* cytosolic proteins, as determined by both cleavable and non-cleavable search approaches applied to the same data set^*a*^

Detection methods	Cleavable		Non-cleavable		Combined (shared)	
FDR	<1%	<5%	<1%	<5%	<1%	<5%
CSM (total)	12,993	18,692	15,955	16,050	n.a.	n.a.
Intralinks	11,856	16,526	15,523	15,523	n.a.	n.a.
Interlinks	1,137	2,166	432	527	n.a.	n.a.
Crosslinks (unique R-R)	2,350	3,290	2,443	2,662	3,525 (1,268)	4,310 (1,642)
Intralinks	2,159	3,043	2,384	2,590	3,308 (1,235)	4,033 (1,600)
Interlinks	191	247	59	72	217 (33)	277 (42)
PPI	157	181	50	59	171 (36)	196 (44)

^*a*^ The combined use of these search strategies resulted in a 54–92% increase in the detection of unique residue-to-residue interactions. Abbreviations: FDR, false discovery rate, CMS, crosslink spectra match; PPI, protein-protein interactions, n.a., not applicable.

### Evaluating PPIs

Our combined interactome analysis generated networks with 261 nodes and 196 edges at FDR of <5%, and 229 nodes and 171 edges at FDR of <1%, respectively. Figure 1B displays the networks with unique PPIs and genes are colored based on hyperLOPIT localization assignments. There are several distinct networks of the interactome. As expected for cytosolic proteome, the most prominent complexes are the abundant 60S ribosome and 40S ribosome complexes comprizing more than 50 proteins. The 19S and 20S proteasome complexes form two separate networks, reflecting the way the proteasomes assemble this structure. The eukaryotic initiation factor 3 (eIF3) complex is also detected. Strikingly, seven dense granular proteins form a densely connected subnetwork that has a connection to the SRS protein network. This indicates that the crosslinking MS can be used for proteins enclosed in membranous secretory vesicles inside the cytosol due to the membrane permeability of the crosslinker.

To validate the feasibility of the interactomes that were generated by the crosslinking data, we compared our findings with other independently assessed large-scale proteomics data, namely subcellular localization by hyperLOPIT (5) and protein complexes by co-elution ToxoNET (34). Of all pairs found in independent data set, excluding missing values, 71.6% of PPIs are in the same location in hyperLOPIT and 61.4% are in the same cluster in ToxoNET (Table 2). The high rate of agreement, relative to disagreement and in comparison to random pairs, indicates that these protein pairs are likely to represent genuine interactions. We further evaluated the concordance among additional biological features—such as gene ontology (GO) components, functions, processes, EC numbers, Pfam IDs, and STRING pairs—and observed a similar trend: high-confidence hits exhibited greater agreement, while low-confidence hits showed reduced concordance.

**TABLE 2:** Summary of the percentage agreement between crosslinked pairs and multiple independent biological data sets^*a*^

Confidence	FDR %	HyperLOPIT	ToxoNET	GO component	GO function	GO process	EC number	PFam ID	STRING
High	<1	71.6	61.4	83.9	77.4	77.8	63.6	25.4	97.4
Medium	1–5	52.9	75.0	88.9	54.5	50.0	66.7	31.3	69.2
Low	5–10	15.8	100	62.5	57.9	50.0	14.3	12.0	63.6
Marginal	10–30	34.9	66.6	67.6	42.9	41.2	35.7	7.1	61.9
Lowest	>30	11.0	6.2	13.0	11.6	3.1	1.6	0.5	13.1
Random	n.a.	10.0	3.1	10.4	8.9	2.3	1.1	0.4	6.9

^*a*^ Percentages are stratified by crosslink confidence level, highlighting that crosslinks with higher confidence exhibit greater agreement. Crosslinked pairs with missing data were excluded from these calculations. Abbreviations: FDR, false discovery rate, n.a., not applicable.

### Improving crosslinking using machine learning

**Fig 2:**
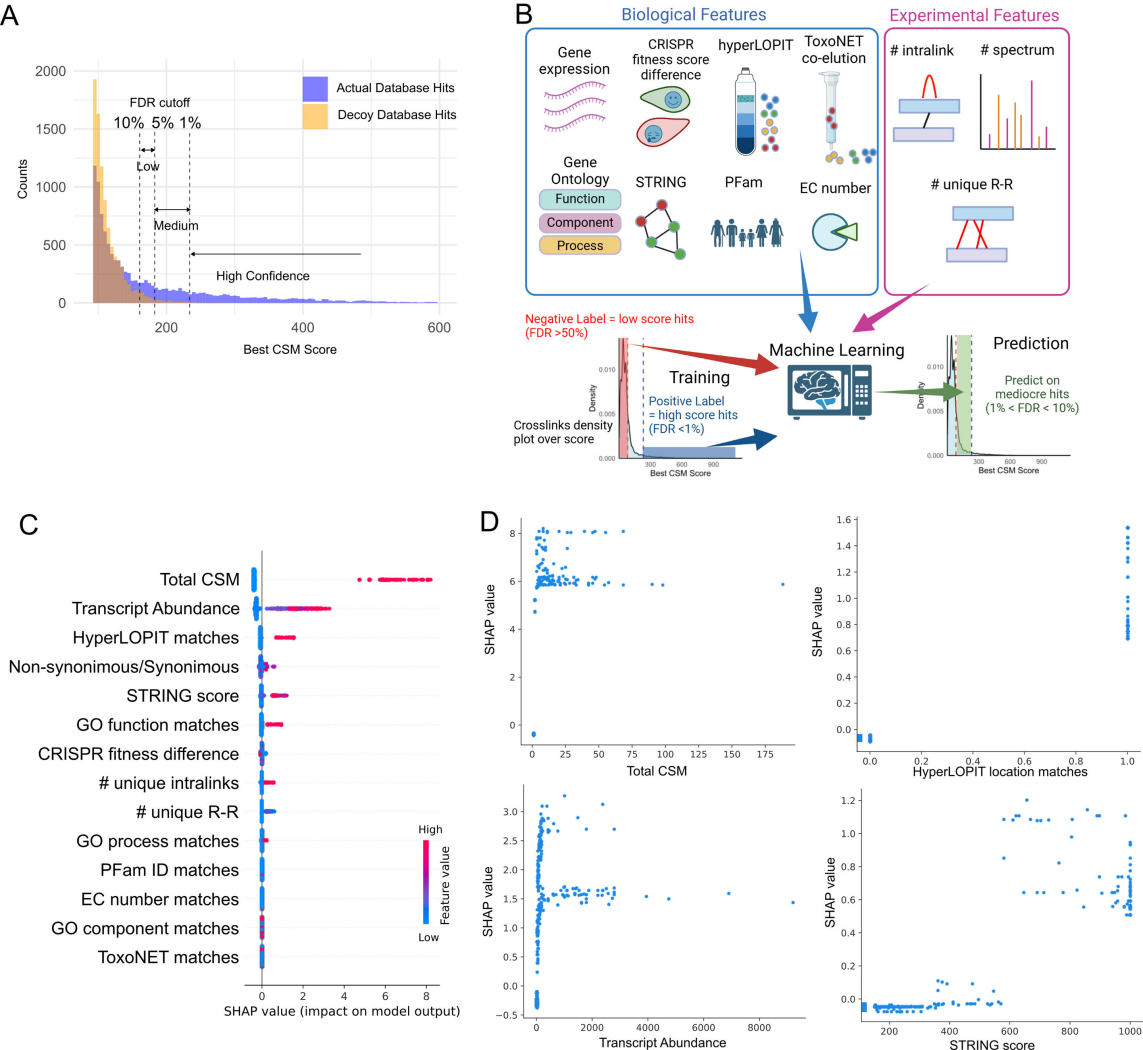
Machine learning. (**A**) Histogram of identification score (best CSM score) in true hits (blue) and decoy hits (yellow). The histogram illustrates the theoretical true hits within the medium- and low-confidence regions, with the confidence cutoff thresholds indicated by dotted lines. The blue areas in the low- and medium-confidence regions, located above the overlap with decoy hits, represent the theoretical true hits that can be salvaged. (**B**) Schematic illustrating the recovery of medium- to low-confidence hits using machine learning based on biological and experimental features. (**C**) Analysis of feature importance and individual prediction contributions in the model. SHapley Additive exPlanations (SHAP) values are presented, with positive values indicating that the feature increases the prediction and negative values suggesting a decrease. Features are ranked from most to least influential. Feature values are color-scaled from high (red) to low (blue), with missing values shown in gray. (**D**) Relationship between feature importance and individual features. A higher total number of CSMs, transcript abundance, STRING scores, and matching hyperLOPIT assignments contribute to positive predictions (as indicated by SHAP values), whereas lower values drive negative predictions during model construction.

A FDR threshold of less than 1% is routinely applied to classify mass spectra as “high confidence,” and 5% is commonly used as “medium confidence” ensuring the reliability of spectral quality. However, an analysis of the histogram depicting spectral hits matched to both the target and decoy (reversed sequence) databases reveals that a substantial proportion of hits within higher FDR ranges are likely true positives. Specifically, 86% of medium-confidence hits (1% < FDR < 5%) and 64% of low-confidence hits (5% < FDR < 10%) are expected to be theoretically genuine (Fig. 2A). Further investigation of these medium- and low-confidence PPIs has enabled the identification of highly probable crosslinks, including the proteins that are known to be in the same complex, such as numerous ribosomal proteins, proteasomal proteins, elongation factor 1 (EF1) complex, eIF3 complex, and prefoldin complex. A higher agreement percentage among the high-confidence hits (Table 2), compared to those of low or random pairs, suggests that we can leverage our prior biological knowledge to effectively distinguish potential true hits from false ones. Consequently, we attempted to salvage these middle- to low-score hits by applying biological features through machine learning algorithms (Fig. 2B). We selected readily available biological features from ToxoDB, which include tachyzoite transcriptomic data, CRISPR fitness scores, hyperLOPIT, Gene Ontology, PFam, and EC numbers. Additionally, we incorporated interactions from the STRING database and experimentally generated ToxoNET data.

In addition to the biological features, recent publications have identified ways to improve crosslink identification, taking into account crosslinks based on the presence of corresponding proteins detected in intralinks (intra-type dependent), and the presence of multiple unique residue-residue interlink pairs (inter-dependent) (35, 36). We therefore included similar features that can be easily implemented for machine learning. Those features are the number of unique residue-residue pairs in the PPI and the number of intralink spectra (Fig. 2B).

We chose a gradient-boosting decision-tree algorithm LightGBM (28) because of its ability to use tabulated data containing both categorical and numerical features, as well as its native support for missing values in binary classification tasks. High-confidence pairs (FDR < 1%) were used as the positive training data, while the lowest-confidence pairs (FDR > 50%) served as negative training data. The resulting model was subsequently evaluated on an independent test set, achieving an accuracy of 0.9905 and a receiver operating characteristic area under the curve (ROC AUC) of 0.9996. One of the key advantages of a decision-tree-based algorithm is the interpretability of the features used in model construction. SHapley Additive exPlanation (SHAP) (37) was used to quantify the contribution of each feature to the model’s predictions. Not surprisingly, the total number of CSMs emerged as the most influential feature, with higher counts strongly predicting true hits (Fig. 2C and D). Additionally, the number of unique intralinks (with a minimum of the pair) was found to be a strong predictor of interaction, which is consistent with recent publications (36). In contrast, the number of unique residue-to-residue interactions did not contribute significantly to the model—likely because most interactions were represented by a single interaction, resulting in insufficient variability for effective prediction.

Biological features also contributed significantly to the construction of the predictive model. One of the most influential biological features was the transcript abundance (minimum of the pair) at the tachyzoite stage. This observation is consistent with our use of tachyzoites for sample preparation, since proteins with high transcript levels generally generate stronger signals in mass-spectrometric assays. The binary features such as the agreement of HyperLOPIT subcellular locations also clearly influence positive prediction (Fig. 2C and D).

Furthermore, the STRING score, which reflects the confidence of interaction based on aggregated experimental data and inferences from protein homology, exhibits a clear positive correlation with the predictions, especially when higher scores (red) were observed (Fig. 2C). Conversely, lower scores (blue) are associated with negative predictions. A detailed analysis of SHAP value and STRING score (Fig. 2D) shows that the threshold for the feature contribution is in the medium confidence range with the STRING score of 600. Although the number of positive and negative instances for the GO match feature was lower than that for other features (Fig. 2C), it still strongly influenced the prediction of true interactions. Collectively, these results demonstrate that incorporating robust large-scale biological features can enable the construction of prediction models capable of effectively evaluating mediocre hits and estimating the probability of true interactions. Overall, use of this approach resulted in an improved interactome which incorporated 33 new PPIs at low confidence (5% < FDR < 10%) and 22 new PPIs at marginal confidence (10% < FDR < 30%) based on the predictions. Improved interactome with detailed information is provided in the Fig. S2. All PPIs are available in File S2.

### Structural mapping

#### Ribosome

**Fig 3:**
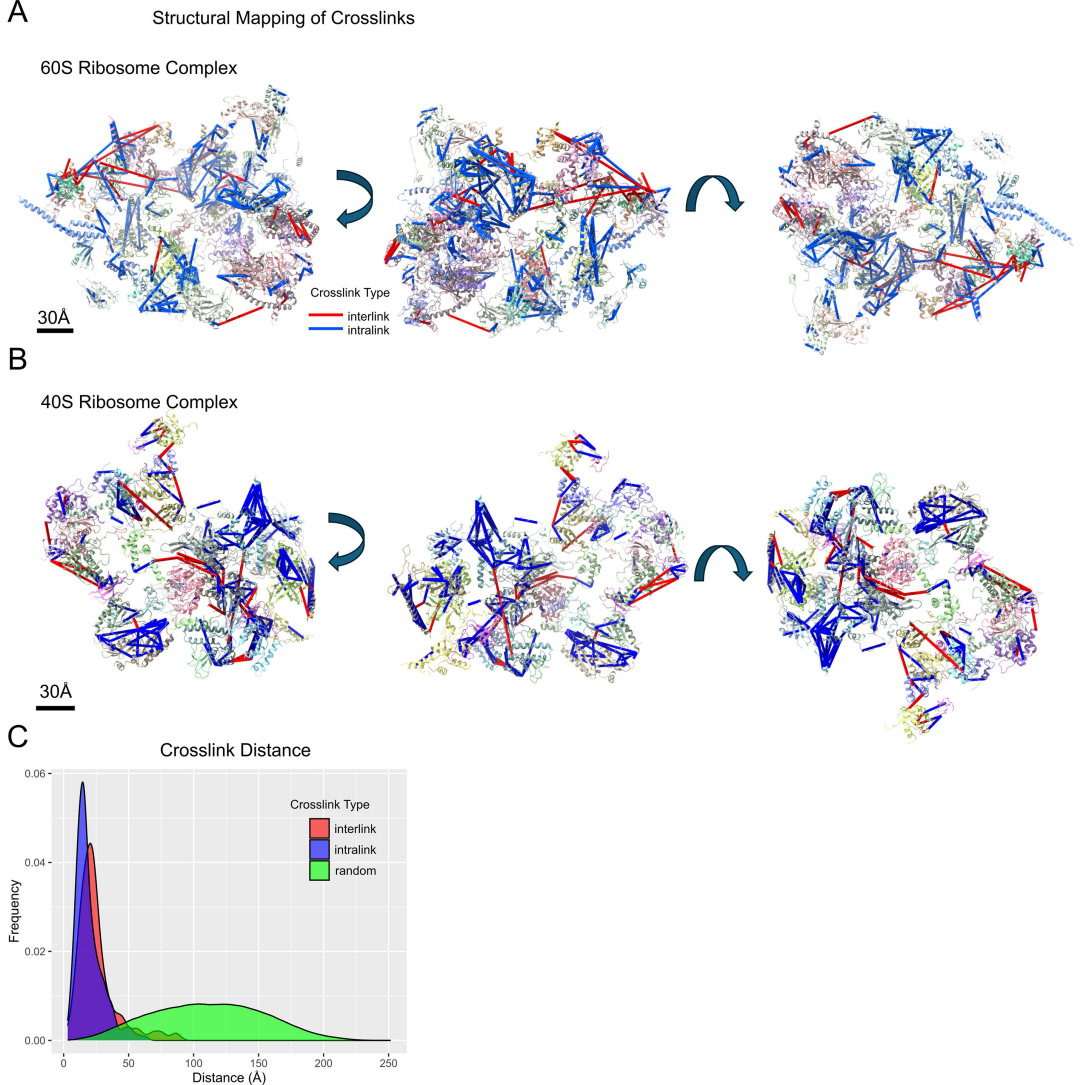
Structural mapping of ribosome crosslinks. Interlinks (red) and intralinks (blue) were mapped onto previously determined cryo-EM structures of the 60S ribosome subunit complex (**A**) and the 40S complex (**B**), with three rotations shown for each structure. (**C**) A frequency histogram depicting the interlinks (red, *M* = 21.8 Å, *n* = 67), intralinks (blue, *M* = 16.5 Å, *n* = 418), and random KSTY-KSTY pairs (green, *M* = 111.0 Å, *n* = 2,324,504). This highlights the specificity of crosslinks relative to random pairs.

To validate the reliability of our crosslinking data, we performed structural mapping of crosslinked residue pairs onto the experimentally determined 3D structures (Fig. 3A and B; Movies S1 and S2). Specifically, we utilized the cryo-EM structure of the 60S ribosomes (PDB: 5XXB) and 40S ribosomes (PDB: 5XXU), which had been previously resolved to near-atomic resolutions (38). For the two ribosome complexes, we mapped and measured the Cα-Cα distances of unique residue-residue inter- and intra-links, with a FDR of less than 5%. Although DSBSO’s nominal spacer arm is 14 Å, inclusion of lysine side-chain extensions and backbone flexibility increases the effective Cα–Cα crosslinking distance to up to ~35 Å (17, 39). Our analysis revealed that the median distances for inter-link were 21.8 Å (*n* = 67) and for intra-link were 16.5 Å (*n* = 418). In contrast, the median distance for all possible KSTY residue pairs (the amino acids that could be linked by the crosslinker) was 111.0 Å (*n* = 2,324,504), thereby demonstrating the validity of the crosslinked pairs (Fig. 3C).

**Fig 4:**
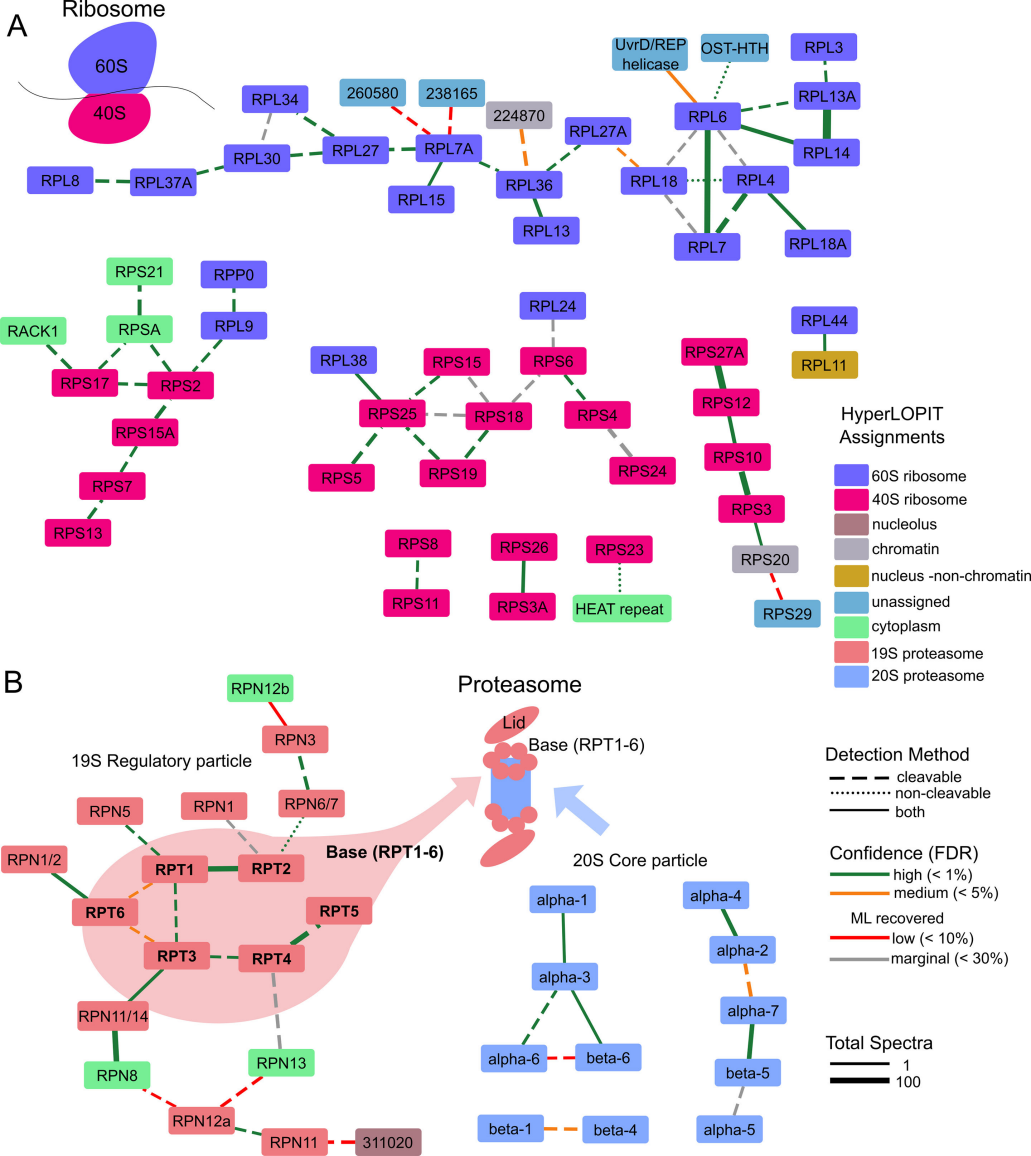
Ribosome and proteasome interactomes. The interactome for (**A**) ribosome-associated proteins and (**B**) proteasome proteins, with nodes color-coded by hyperLOPIT assignments. The network edges are color-coded based on confidence, with line styles indicating detection methods and thickness representing the total number of spectra for each interaction. These networks also integrate salvaged interactions identified through machine learning.

Due to weak local density and its transient association with the ribosome, the cryo-EM reconstruction did not resolve signaling scaffold protein RACK1 (TGME49_216880, annotated as POC1 centriolar protein in ToxoDB) within the 40S subunit. In contrast, our crosslinking data (Fig. 4A) reveals that RACK1 interacts with RPS17, demonstrating that XL-MS can detect transient associations. Additionally, various ribosomal proteins (RPS20, RPS21, RPS29, PRSA, RPL11, and RACK1) that were mischaracterized with HyperLOPIT data as cytosolic, chromatin, nucleus (non-chromatin), or unassigned are directly connected to the networks. This finding underscores the ability of XL-MS to complement and refine data obtained from other mainstream approaches. This data also demonstrates that XL-MS not only identified known protein interactions but also found potential novel interactions. For example, the HEAT-repeat containing protein (TGME49_231600), which has homology to importin β, is crosslinked to ribosomal protein RPS23. This putative importin β might transfer nascent cytosolic RPS23 into the nucleus to be assembled with ribosomal RNA at the nucleolus as described in other organisms (40). As depicted in Fig. 4, the incorporation of low-confidence (red lines, 5% < FDR < 10%) and marginal-confidence (gray lines, 10% < FDR < 30%) hits recovered by the machine learning model aligns well with the other hits and augments construction of the interactomes.

### Proteasome complex

The proteasome consists of two major complexes: the 20S core particles, which function as the proteolytic shredder, and 19S regulatory particles. Our observations (Fig. 4B) reveal a distinct segregation between these two assemblies. The 19S regulatory particle is further organized into two subcomplexes, the Lid and the Base. The Base comprises six AAA+ ATPases (Rpt1 through Rpt6; Fig. 4B, Bold) that form a ring in a defined sequential order (41). Our crosslinking data not only confirms the ring structure but also corroborates the specific sequence of the ATPase subunits. Consistent with the ribosome findings, our XL-MS data revealed that three proteasomal proteins—RPN8, RPN12b, and RPN13—which were misclassified as cytosolic based on HyperLOPIT, are directly associated with the 20S core particle. These findings clarify the distinct structural organization of proteasome assemblies and reveal new functional associations, thereby enhancing our understanding of proteasomal regulation and protein degradation.

### Homo-dimer detection

Crosslinks involving peptides that map to the same residues and share identical or overlapping sequences provide evidence that two copies of the same protein reside in close spatial proximity, indicative of homo-dimer formation. A thorough analysis of the peptide sequences identified 101 proteins that exhibited homo-dimer crosslinks, of which 16 had 3 or more links (Table 3). Although direct evidence of homo-dimerization in *T. gondii* is scarce, many homologs in other organisms are known to form homodimers, such as peroxiredoxin 1 (42), phosphoenolpyruvate (PEP) carboxykinase I (43), coronin (44), fructose-1,6-bisphosphate aldolase (45), all of which are highly represented in the data set. These findings strongly support the validity of dimerization detection using cross-link mass spectrometry data.

**TABLE 3:** A list of homodimers detected from the crosslinking data, including only proteins with homo-dimer crosslinking peptides containing at least three unique residue-to-residue interactions (RRIs)^*a*^

Gene	Description	#RRI
TGME49_209030	Actin ACT1	7
TGME49_203310	GRA7	5
TGME49_217890	Peroxiredoxin PRX1	4
TGME49_227620	GRA2	4
TGME49_289650	PEP-carboxykinase I	4
TGME49_205320	Hypothetical	3
TGME49_216970	Coronin	3
TGME49_221470	Hypothetical	3
TGME49_227280	GRA3	3
TGME49_236040	Gructose-1,6-bisphosphate aldolase	3
TGME49_249390	Glutamate/leucine/phenylalanine/valine dehydrogenase family protein	3
TGME49_273490	Glutamine synthetase, type I	3
TGME49_286420	Elongation factor 1-alpha (EF-1-ALPHA)	3
TGME49_290670	Leucyl aminopeptidase LAP	3
TGME49_293740	Hypothetical	3
TGME49_295350	Nucleoside diphosphate kinase	3

^*a*^ False detections due to tandem repeats have been removed.

**Fig 5:**
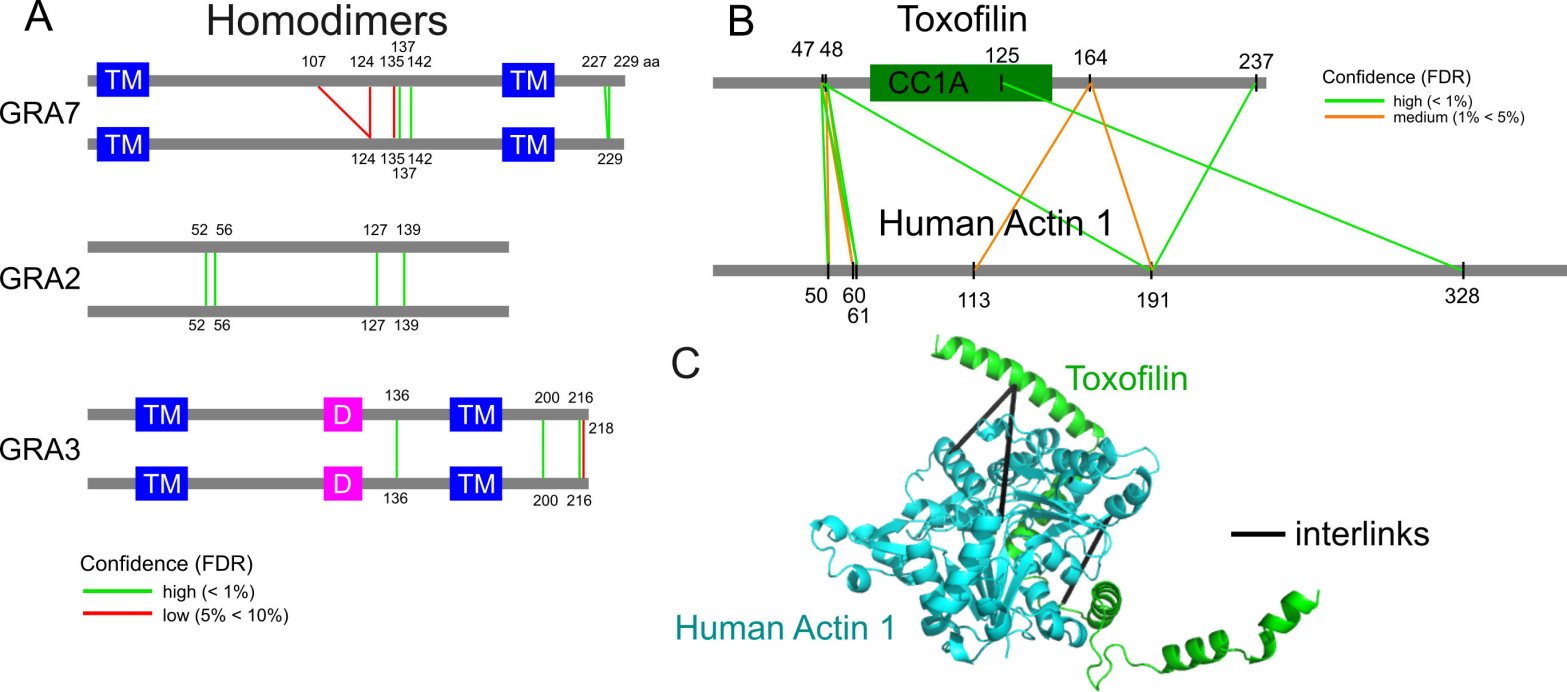
Protein interactions. (**A**) Representative homodimers determined by crosslinking data are shown with protein amino acid positions and domain lengths drawn to scale (TM: transmembrane domain, D: dimerization domain). The lines indicate the residues involved in dimerization. (**B**) This panel depicts a representative human–parasite interaction at the residue-to-residue level, highlighting the CC1A domain in toxofilin, which is required for actin binding. (**C**) Structural mapping of toxofilin-actin crosslinks is presented on a 3D model based on homology modeling of a crystallographically determined structure (PDB: 2Q97) that accounts for the SNP present in toxofilin.

Interestingly, several GRA proteins were identified among the homodimer candidates, including GRA7, GRA2, GRA3, GRA32, GRA8, and GRA6. Specifically, GRA7 exhibited five unique residue pairs for homo-dimerization. Mapping the crosslinks revealed potential hotspots for dimerization (Fig. 5A). Notably, GRA7 is known to form complexes with ROP18/ROP5 and to alter the oligomerization of Irga6 (46, 47). Similarly, GRA3 demonstrated three unique homodimerization residue pairs and is previously recognized for its capacity to dimerize, interact with the host Golgi apparatus, and facilitate membrane scavenging (48). These findings suggest that GRA protein dimerization may play a critical role in immune evasion and the appropriation of host cellular functions.

### Host-parasite interactions

To evaluate the potential crosslinking events between *T. gondii* and human proteins, we re-analyzed our data set using a combined database containing protein sequences from both organisms. Since the samples were prepared from purified parasite cytosolic lysates, which were intentionally depleted of host-derived material, very few human–parasite PPIs were identified. The most prominent pair observed was the interaction between toxofilin (TGME49_214080) and actin with eleven unique residue-to-residue crosslinks (Fig. 5B). Toxofilin is known to bind to globular actin via its CC1A domain (49). Although several peptides were mapped to both human and parasite actin, other peptides were unique to the human sequence, demonstrating the potential for dissecting human-parasite interactions. Three crosslinks were mapped onto the structure, which was homology modeled using the portion of toxofilin crystallized with human actin 1 (PDB: 2Q97), indicating that these crosslinks are consistent with the previously characterized host-pathogen complex (Fig. 5C).

### GRA network

Unexpectedly, our study identified the dense granule protein network as a prominent subnetwork. This finding is noteworthy because the sample preparation was designed to process only cytosolic proteins. Considering that the majority of dense granule proteins are secreted into parasitophorous vacuoles, their presence in our samples likely occurs in small amounts, predominantly within dense granule membranous vesicles that “contaminated” the cytosolic preparation.

**Fig 6:**
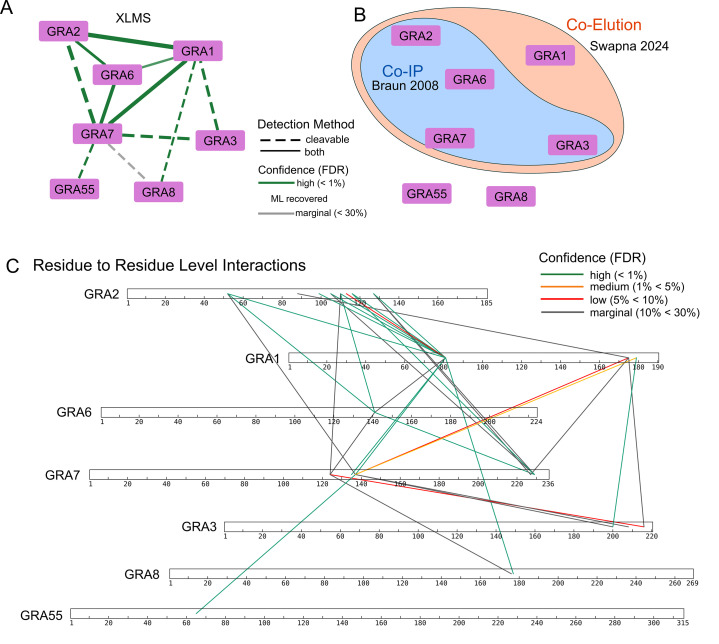
Dense granule (GRA) protein interactions identified by cross-linked mass spectrometry. (**A**) GRA interactome based on crosslinking data. (**B**) GRA interactome derived from previous studies using co-immunoprecipitation with GRA2 (50) and large-scale co-elution study (ToxoNET) (6). (**C**) The GRA interactome presented at the residue-to-residue level, drawn to scale, with lines representing interlinks.

Figure 6A presents the selected interactome of known GRAs. Previous studies have established that GRA proteins within dense granules form oligomeric complexes exceeding 1 MDa throughout the secretory pathway (50). These pre-assembled oligomeric GRA complexes are thought to hide the hydrophobic domains, thus maintaining solubility in the dense granule matrix. Previous co-immunoprecipitation experiments (50) have demonstrated that GRA2 associates with GRA3, GRA6, and GRA7 (Fig. 6B). Our crosslinking data further distinguishes between direct and indirect binding interactions. Notably, the interaction between GRA2 and GRA3 occurs indirectly through GRA7 (Fig. 6A), offering intricate insights into complex assembly that were previously inaccessible through conventional assays.

A more recent study examining the co-elution of native complexes in ToxoNET (6) produced a similar set of GRA proteins as potential complex components. In particular, ToxoNET Cluster #26 comprises GRA1, GRA2, GRA3, GRA4, GRA6, GRA7 (Fig. 6B), and additional microneme proteins. Our data unambiguously delineate the specific binding relationships among these proteins.

To enhance the resolution of the complex, individual residue-to-residue interactions were mapped for each protein (Fig. 6C). GRA2, for instance, is characterized by a central domain (88–127) that serves as a hotspot, engaging 12 unique interactions with GRA1, GRA6, and GRA7. Although GRA2 was previously considered the hub of this complex, our interactome analysis indicates that GRA7 exhibits a greater number of connections, thereby possibly functioning as the central hub. These findings underscore the utility of XL-MS in elucidating complex, large-scale PPIs.

## DISCUSSION

The current study demonstrates that XL-MS can effectively map the complex network of PPIs within the cytosolic fraction of *T. gondii*. By integrating both MS-cleavable and non-cleavable spectra, we identified thousands of residue–residue contacts that validated known assemblies, such as ribosomal and proteasomal complexes, and uncovered novel interactions, particularly among dense granule proteins. Structural mapping further confirmed the spatial feasibility of the observed crosslinks, while a machine learning model leveraging diverse biological features salvaged interactions from mediocre-score hits by distinguishing true associations from potential false positives. Overall, these results reinforce the robustness of XL-MS as a tool for interactome analysis and provide fresh insights into the molecular architecture and functional dynamics of *T. gondii*.

Crosslinker concentrations approaching the solubility limit (5 mM) and extended incubation periods (2 h for crosslinking; overnight for click chemistry) were used to maximize the yield of crosslinked spectra, with the acknowledged trade-off of increased nonspecific interactions. However, optimal conditions must be determined empirically for each system. As a guideline, we recommend beginning optimization with crosslinker concentrations of 0.5–5 mM, crosslinking incubation times of 30 min to 2 h, and click-chemistry incubations ranging from 1 h to overnight. Beyond optimizing the reaction conditions, one of the greatest challenges was sourcing high-quality DSBSO. ^1^H-NMR of multiple DSBSO batches from different suppliers revealed that even subtle NHS-ester hydrolysis markedly reduced crosslinking efficiency and resulted in fewer identifiable crosslinks.

A limitation of our experimental design is that we performed no additional fractionation beyond cytosol extraction. This restriction limits the majority of detection to highly abundant proteins—often estimated to represent roughly the top 20% of the proteome—due to their high dynamic range (51). Incorporating further subcellular fractionation techniques, such as hyperLOPIT, or affinity purification methods like immunoprecipitation or BioID, would significantly enhance proteomic coverage by distributing and resolving these abundant proteins, allowing better detection of proteins of lower abundance.

Circularity arises when features encoding known interactions (e.g., STRING scores and hyperLOPIT localization) bias our model, elevating low-quality spectra linked to well-characterized proteins (Fig. 2C and D) and enriching familiar complexes regardless of spectral evidence. To counter this, future work should employ orthogonal validation and stringent negative controls to preserve genuine low-confidence crosslink recovery and sensitivity to novel interactions.

Because the crosslinker’s spacer arm extends up to 14 Å, detecting a crosslink between two proteins does not necessarily imply a direct interaction; distinct assemblies held within 14 Å by other proteins or macromolecules may also crosslink efficiently. Recognizing this distinction is particularly crucial when studying poorly characterized proteins such as the GRAs.

The development of this crosslinking mass spectrometric protocol will allow additional investigations of the various protein interactomes in *T. gondii*. For example, the capacity of this parasite to persist within the host over extended periods is a key factor in its transmission and ability to cause recrudescent disease in immunocompromised hosts. The cyst wall, which serves as the interface between the cyst and the host, plays a critical role in the pathogenesis of chronic toxoplasmosis. Until recently, it was impossible to maintain the bradyzoite culture for long periods of time in tissue culture and develop a structurally distinct thick cyst wall layer present in the cysts from mouse brains. The use of myotubes as a host enabled the development of large mature cysts (28 days post-infection) with thick walls that developed broad tolerance to various antiparasitic drugs as well as acid/pepsin treatment (52). The ability to generate mature cysts using *in vitro* systems, instead of isolating mouse-derived brain cysts, will facilitate the use of XL-MS to study the composition and protein interactome of the *T. gondii* cyst wall.

While our crosslinking studies have identified numerous potential interactions, including those between parasite and human proteins, these findings will require further experimental validation. Future investigations employing complementary techniques, such as co-immunoprecipitation, Y2H assays, or Förster resonance energy transfer, should be undertaken to confirm the predicted interactions and further elucidate their functional significance.

## Data Availability

Proteomic data sets were deposited to ProteomeXchange under the project accession number PXD062479 (https://www.ebi.ac.uk/pride/archive/projects/PXD062479) and to ToxoDB (EuPATHdB; https://toxodb.org/toxo/app). The data are fully available without any restrictions.

## SUPPLEMENTAL MATERIAL

File S1A mbio.02159-25-s0001.txt R with the tidyverse package scripts utilized for crosslink processing and visualization.

File S1B mbio.02159-25-s0002.txt Python with LightGBM scripts utilized for crosslink processing and visualization.

File S2 mbio.02159-25-s0003.xlsx GO term analysis of the crosslinked proteins.

File S3 mbio.02159-25-s0004.xlsx Comprehensive PPI table with LightGBM prediction.

File S4 mbio.02159-25-s0005.xlsx Comprehensive RRI table.

Figure S1 mbio.02159-25-s0006.pdf SEC fractions.

Figure S2 mbio.02159-25-s0007.pdf *T. gondii* protein interactome.

Legends mbio.02159-25-s0008.docx Legends for all supplemental files.

Movie S1 mbio.02159-25-s0009.mp4 Crosslinks mapped onto the 60S large ribosomal subunit (PDB: 5XXB).

Movie S2 mbio.02159-25-s0010.mp4 Crosslinks mapped onto cryo-EM structures of the 40S small ribosomal subunit (PDB: 5XXU).

ASM does not own the copyrights to Supplemental Material that may be linked to, or accessed through, an article. The authors have granted ASM a non-exclusive, world-wide license to publish the Supplemental Material files. Please contact the corresponding author directly for reuse.

## References

[1] Molan A, Nosaka K, Hunter M, Wang W. 2019. Global status of Toxoplasma gondii infection: systematic review and prevalence snapshots. Trop Biomed 36:898–925. 33597463

[2] Wang Z-D, Wang S-C, Liu H-H, Ma H-Y, Li Z-Y, Wei F, Zhu X-Q, Liu Q. 2017. Prevalence and burden of Toxoplasma gondii infection in HIV-infected people: a systematic review and meta-analysis. Lancet HIV 4:e177–e188. doi:10.1016/S2352-3018(17)30005-X 28159548

[3] Vidal JE. 2019. HIV-related cerebral toxoplasmosis revisited: current concepts and controversies of an old disease. J Int Assoc Provid AIDS Care 18:2325958219867315. doi:10.1177/2325958219867315 31429353 PMC6900575

[4] Choi CP, Moon AS, Back PS, Jami-Alahmadi Y, Vashisht AA, Wohlschlegel JA, Bradley PJ. 2019. A photoactivatable crosslinking system reveals protein interactions in the Toxoplasma gondii inner membrane complex. PLoS Biol 17:e3000475. doi:10.1371/journal.pbio.3000475 31584943 PMC6795473

[5] Barylyuk K, Koreny L, Ke H, Butterworth S, Crook OM, Lassadi I, Gupta V, Tromer E, Mourier T, Stevens TJ, Breckels LM, Pain A, Lilley KS, Waller RF. 2020. A comprehensive subcellular atlas of the Toxoplasma proteome via hyperLOPIT provides spatial context for protein functions. Cell Host Microbe 28:752–766. doi:10.1016/j.chom.2020.09.011 33053376 PMC7670262

[6] Swapna LS, Stevens GC, Sardinha-Silva A, Hu LZ, Brand V, Fusca DD, Wan C, Xiong X, Boyle JP, Grigg ME, Emili A, Parkinson J. 2024. ToxoNet: a high confidence map of protein-protein interactions in Toxoplasma gondii. PLoS Comput Biol 20:e1012208. doi:10.1371/journal.pcbi.1012208 38900844 PMC11219001

[7] Kaake RM, Wang X, Burke A, Yu C, Kandur W, Yang Y, Novtisky EJ, Second T, Duan J, Kao A, Guan S, Vellucci D, Rychnovsky SD, Huang L. 2014. A new in vivo cross-linking mass spectrometry platform to define protein-protein interactions in living cells. Mol Cell Proteomics 13:3533–3543. doi:10.1074/mcp.M114.042630 25253489 PMC4256503

[8] Rey M, Dhenin J, Kong Y, Nouchikian L, Filella I, Duchateau M, Dupré M, Pellarin R, Duménil G, Chamot-Rooke J. 2021. Advanced in vivo cross-linking mass spectrometry platform to characterize proteome-wide protein interactions. Anal Chem 93:4166–4174. doi:10.1021/acs.analchem.0c04430 33617236

[9] Gao H, Zhao Q, Gong Z, Zhong B, Chen J, Sui Z, Li X, Liang Z, Zhang Y, Zhang L. 2022. Alkynyl-enrichable carboxyl-selective crosslinkers to increase the crosslinking coverage for deciphering protein structures. Anal Chem 94:12398–12406. doi:10.1021/acs.analchem.2c02205 36031802

[10] Sun N, Wang Y, Wang J, Sun W, Yang J, Liu N. 2020. Highly efficient peptide-based click chemistry for proteomic profiling of nascent proteins. Anal Chem 92:8292–8297. doi:10.1021/acs.analchem.0c00594 32434323

[11] Matzinger M, Kandioller W, Doppler P, Heiss EH, Mechtler K. 2020. Fast and highly efficient affinity enrichment of azide-A-DSBSO cross-linked peptides. J Proteome Res 19:2071–2079. doi:10.1021/acs.jproteome.0c00003 32250121 PMC7199212

[12] Sinn LR, Giese SH, Stuiver M, Rappsilber J. 2022. Leveraging parameter dependencies in high-field asymmetric waveform ion-mobility spectrometry and size exclusion chromatography for proteome-wide cross-linking mass spectrometry. Anal Chem 94:4627–4634. doi:10.1021/acs.analchem.1c04373 35276035 PMC8943524

[13] Jiao F, Yu C, Wheat A, Wang X, Rychnovsky SD, Huang L. 2022. Two-dimensional fractionation method for proteome-wide cross-linking mass spectrometry analysis. Anal Chem 94:4236–4242. doi:10.1021/acs.analchem.1c04485 35235311 PMC9056026

[14] Schnirch L, Nadler-Holly M, Siao S-W, Frese CK, Viner R, Liu F. 2020. Expanding the depth and sensitivity of cross-link identification by differential ion mobility using high-field asymmetric waveform ion mobility spectrometry. Anal Chem 92:10495–10503. doi:10.1021/acs.analchem.0c01273 32643919

[15] Stieger CE, Doppler P, Mechtler K. 2019. Optimized fragmentation improves the identification of peptides cross-linked by MS-cleavable reagents. J Proteome Res 18:1363–1370. doi:10.1021/acs.jproteome.8b00947 30693776

[16] Mayoral J, Tomita T, Tu V, Aguilan JT, Sidoli S, Weiss LM. 2020. Toxoplasma gondii PPM3C, a secreted protein phosphatase, affects parasitophorous vacuole effector export. PLoS Pathog 16:e1008771. doi:10.1371/journal.ppat.1008771 33370417 PMC7793252

[17] Wheat A, Yu C, Wang X, Burke AM, Chemmama IE, Kaake RM, Baker P, Rychnovsky SD, Yang J, Huang L. 2021. Protein interaction landscapes revealed by advanced in vivo cross-linking-mass spectrometry. Proc Natl Acad Sci USA 118:e2023360118. doi:10.1073/pnas.2023360118 34349018 PMC8364181

[18] Wiśniewski JR, Zougman A, Nagaraj N, Mann M. 2009. Universal sample preparation method for proteome analysis. Nat Methods 6:359–362. doi:10.1038/nmeth.1322 19377485

[19] Wiśniewski JR. 2019. Filter aided sample preparation - a tutorial. Anal Chim Acta 1090:23–30. doi:10.1016/j.aca.2019.08.032 31655642

[20] Klössel S, Zhu Y, Amado L, Bisinski DD, Ruta J, Liu F, Montoro AG. 2023. Yeast TLDc domain-containing proteins control assembly and subcellular localization of the V-ATPase. bioRxiv. doi:10.1101/2023.08.21.554079 PMC11066047 38589611

[21] Amos B, Aurrecoechea C, Barba M, Barreto A, Basenko EY, Bażant W, Belnap R, Blevins AS, Böhme U, Brestelli J, et al.. 2022. VEuPathDB: the eukaryotic pathogen, vector and host bioinformatics resource center. Nucleic Acids Res 50:D898–D911. doi:10.1093/nar/gkab929 34718728 PMC8728164

[22] Birklbauer MJ, Matzinger M, Müller F, Mechtler K, Dorfer V. 2023. MS Annika 2.0 identifies cross-linked peptides in MS2-MS3-based workflows at high sensitivity and specificity. J Proteome Res 22:3009–3021. doi:10.1021/acs.jproteome.3c00325 37566781 PMC10476269

[23] Chen Z-L, Meng J-M, Cao Y, Yin J-L, Fang R-Q, Fan S-B, Liu C, Zeng W-F, Ding Y-H, Tan D, Wu L, Zhou W-J, Chi H, Sun R-X, Dong M-Q, He S-M. 2019. A high-speed search engine pLink 2 with systematic evaluation for proteome-scale identification of cross-linked peptides. Nat Commun 10:3404. doi:10.1038/s41467-019-11337-z 31363125 PMC6667459

[24] R Core Team. 2021. R a language and environment for statistical computing. R Foundation for Statistical Computing, Vienna. - References - Scientific Research Publishing. Available from: https://www.scirp.org/reference/referencespapers?referenceid=3131254. Retrieved 05 Mar 2025.

[25] Wickham H, Averick M, Bryan J, Chang W, McGowan L, François R, Grolemund G, Hayes A, Henry L, Hester J, Kuhn M, Pedersen T, Miller E, Bache S, Müller K, Ooms J, Robinson D, Seidel D, Spinu V, Takahashi K, Vaughan D, Wilke C, Woo K, Yutani H. 2019. Welcome to the Tidyverse. JOSS 4:1686. doi:10.21105/joss.01686

[26] Lenz S, Sinn LR, O’Reilly FJ, Fischer L, Wegner F, Rappsilber J. 2021. Reliable identification of protein-protein interactions by crosslinking mass spectrometry. Nat Commun 12:3564. doi:10.1038/s41467-021-23666-z 34117231 PMC8196013

[27] Sidik SM, Huet D, Ganesan SM, Huynh M-H, Wang T, Nasamu AS, Thiru P, Saeij JPJ, Carruthers VB, Niles JC, Lourido S. 2016. A genome-wide CRISPR screen in Toxoplasma identifies essential apicomplexan genes. Cell 166:1423–1435. doi:10.1016/j.cell.2016.08.019 27594426 PMC5017925

[28] Ke G, Meng Q, Finley T, Wang T, Chen W, Ma W, Ye Q, Liu T-Y. 2017. LightGBM: a highly efficient gradient boosting decision tree. Proceedings of the 31st international conference on neural information processing systems, p 3149–3157 Curran Associates Inc, Red Hook, NY, USA

[29] Akiba T, Sano S, Yanase T, Ohta T, Koyama M. 2019. Optuna: a next-generation hyperparameter optimization framework. arXiv. doi:10.48550/arXiv.1907.10902

[30] Shannon P, Markiel A, Ozier O, Baliga NS, Wang JT, Ramage D, Amin N, Schwikowski B, Ideker T. 2003. Cytoscape: a software environment for integrated models of biomolecular interaction networks. Genome Res 13:2498–2504. doi:10.1101/gr.1239303 14597658 PMC403769

[31] Combe CW, Graham M, Kolbowski L, Fischer L, Rappsilber J. 2024. xiVIEW: visualisation of crosslinking mass spectrometry data. J Mol Biol 436:168656. doi:10.1016/j.jmb.2024.168656 39237202

[32] Meng EC, Goddard TD, Pettersen EF, Couch GS, Pearson ZJ, Morris JH, Ferrin TE. 2023. UCSF Chimerax: tools for structure building and analysis. Protein Sci 32:e4792. doi:10.1002/pro.4792 37774136 PMC10588335

[33] Lagerwaard IM, Albanese P, Jankevics A, Scheltema RA. 2022. Xlink mapping and analySis (XMAS) - smooth integrative modeling in ChimeraX. Bioinformatics. doi:10.1101/2022.04.21.489026

[34] Swapna LS, Stevens GC, da Silva AS, Hu LZ, Brand V, Fusca DD, Xiong X, Boyle JP, Grigg ME, Emili A, Parkinson J. 2021. ToxoNet: a high confidence map of protein-protein interactions in Toxoplasma gondii reveals novel virulence factors implicated in host cell invasion. bioRxiv. doi:10.1101/2021.09.14.460186

[35] Bogdanow B, Wang C, Ruwolt M, Ruta J, Mühlberg L, Zeng W, Elofsson A, Liu F. 2023. Enhancing inter-link coverage in cross-linking mass spectrometry through context-sensitive subgrouping and decoy fusion. bioRxiv. doi:10.1101/2023.07.19.549678

[36] Chen X, Sailer C, Kammer KM, Fürsch J, Eisele MR, Sakata E, Pellarin R, Stengel F. 2022. Mono- and intralink filter (mi-filter) to reduce false identifications in cross-linking mass spectrometry data. Anal Chem 94:17751–17756. doi:10.1021/acs.analchem.2c00494 36510358 PMC9798375

[37] Lundberg S, Lee S-I. 2017. A unified approach to interpreting model predictions. arXiv. doi:10.48550/arXiv.1705.07874

[38] Li Z, Guo Q, Zheng L, Ji Y, Xie Y-T, Lai D-H, Lun Z-R, Suo X, Gao N. 2017. Cryo-EM structures of the 80S ribosomes from human parasites Trichomonas vaginalis and Toxoplasma gondii. Cell Res 27:1275–1288. doi:10.1038/cr.2017.104 28809395 PMC5630675

[39] Klössel S, Zhu Y, Amado L, Bisinski DD, Ruta J, Liu F, González Montoro A. 2024. Yeast TLDc domain proteins regulate assembly state and subcellular localization of the V-ATPase. EMBO J 43:1870–1897. doi:10.1038/s44318-024-00097-2 38589611 PMC11066047

[40] Jäkel S, Görlich D. 1998. Importin beta, transportin, RanBP5 and RanBP7 mediate nuclear import of ribosomal proteins in mammalian cells. EMBO J 17:4491–4502. doi:10.1093/emboj/17.15.4491 9687515 PMC1170780

[41] Tomko RJ Jr, Hochstrasser M. 2011. Order of the proteasomal ATPases and eukaryotic proteasome assembly. Cell Biochem Biophys 60:13–20. doi:10.1007/s12013-011-9178-4 21461838 PMC3256250

[42] Lee W, Choi K-S, Riddell J, Ip C, Ghosh D, Park J-H, Park Y-M. 2007. Human peroxiredoxin 1 and 2 are not duplicate proteins: the unique presence of CYS83 in Prx1 underscores the structural and functional differences between Prx1 and Prx2. J Biol Chem 282:22011–22022. doi:10.1074/jbc.M610330200 17519234

[43] McLeod MJ, Holyoak T. 2023. Biochemical, structural, and kinetic characterization of PP_i_ -dependent phosphoenolpyruvate carboxykinase from Propionibacterium freudenreichii. Proteins 91:1261–1275. doi:10.1002/prot.26513 37226637

[44] Spoerl Z, Stumpf M, Noegel AA, Hasse A. 2002. Oligomerization, F-actin interaction, and membrane association of the ubiquitous mammalian coronin 3 are mediated by its carboxyl terminus. J Biol Chem 277:48858–48867. doi:10.1074/jbc.M205136200 12377779

[45] St-Jean M, Izard T, Sygusch J. 2007. A hydrophobic pocket in the active site of glycolytic aldolase mediates interactions with Wiskott-Aldrich syndrome protein. J Biol Chem 282:14309–14315. doi:10.1074/jbc.M611505200 17329259

[46] Etheridge RD, Alaganan A, Tang K, Lou HJ, Turk BE, Sibley LD. 2014. The Toxoplasma pseudokinase ROP5 forms complexes with ROP18 and ROP17 kinases that synergize to control acute virulence in mice. Cell Host Microbe 15:537–550. doi:10.1016/j.chom.2014.04.002 24832449 PMC4086214

[47] Alaganan A, Fentress SJ, Tang K, Wang Q, Sibley LD. 2014. Toxoplasma GRA7 effector increases turnover of immunity-related GTPases and contributes to acute virulence in the mouse. Proc Natl Acad Sci USA 111:1126–1131. doi:10.1073/pnas.1313501111 24390541 PMC3903209

[48] Deffieu MS, Alayi TD, Slomianny C, Tomavo S. 2019. The Toxoplasma gondii dense granule protein TgGRA3 interacts with host Golgi and dysregulates anterograde transport. Biol Open 8:bio039818. doi:10.1242/bio.039818 30814066 PMC6451337

[49] Jan G, Delorme V, David V, Revenu C, Rebollo A, Cayla X, Tardieux I. 2007. The toxofilin-actin-PP2C complex of Toxoplasma: identification of interacting domains. Biochem J 401:711–719. doi:10.1042/BJ20061324 17014426 PMC1770844

[50] Braun L, Travier L, Kieffer S, Musset K, Garin J, Mercier C, Cesbron-Delauw M-F. 2008. Purification of Toxoplasma dense granule proteins reveals that they are in complexes throughout the secretory pathway. Mol Biochem Parasitol 157:13–21. doi:10.1016/j.molbiopara.2007.09.002 17959262

[51] Picotti P, Bodenmiller B, Mueller LN, Domon B, Aebersold R. 2009. Full dynamic range proteome analysis of S. cerevisiae by targeted proteomics. Cell 138:795–806. doi:10.1016/j.cell.2009.05.051 19664813 PMC2825542

[52] Christiansen C, Maus D, Hoppenz E, Murillo-León M, Hoffmann T, Scholz J, Melerowicz F, Steinfeldt T, Seeber F, Blume M. 2022. In vitro maturation of Toxoplasma gondii bradyzoites in human myotubes and their metabolomic characterization. Nat Commun 13:1168. doi:10.1038/s41467-022-28730-w 35246532 PMC8897399

