# Supplementary material for: Mapping a Toxoplasma gondii interactome by crosslinking mass spectrometry and machine learning
Source: mBio. 2025 Aug 28;16(10):e02159-25. doi: 10.1128/mbio.02159-25 (PMC12505969; doi:10.1128/mbio.02159-25)
Supplement: Figure S1 — SEC fractions. [file mbio.02159-25-s0006.pdf]

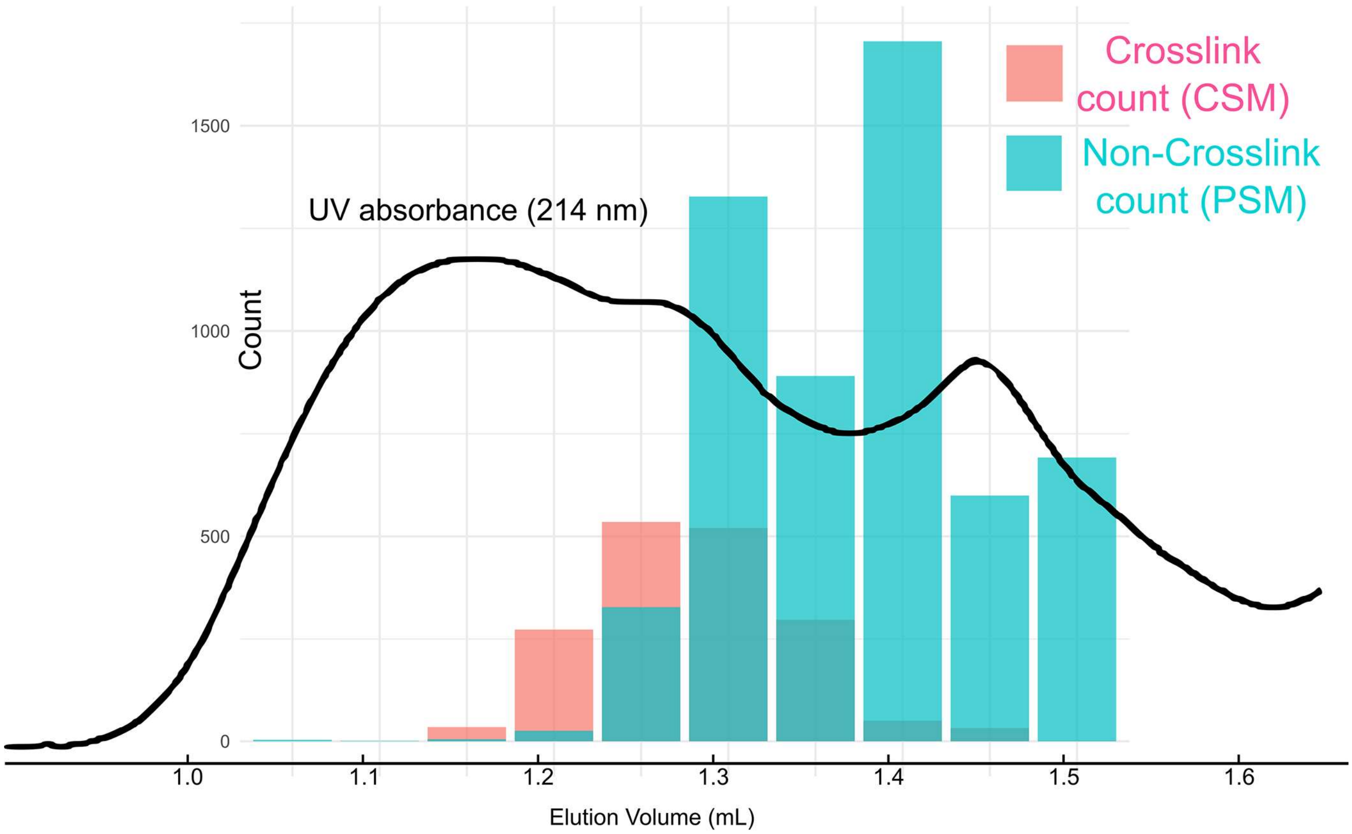

Supplemental Figure S1. SEC Fractions
The number of crosslinks (CSM) and non-crosslinks (PSM) identified in the each fractions after SEC fractionation. The fraction from 1.18 ml to 1.23 ml had highest ratio (46.6) of corsslink/non-crosslink while the fraction from 1.23 ml to 1.28 ml had highest number (535) of crosslinks. The trace is the UV absorbance at 214 nm representing the amount of peptides.
