# Supplementary material for: Mapping a Toxoplasma gondii interactome by crosslinking mass spectrometry and machine learning
Source: mBio. 2025 Aug 28;16(10):e02159-25. doi: 10.1128/mbio.02159-25 (PMC12505969; doi:10.1128/mbio.02159-25)
Supplement: Figure S2 — T. gondii protein interactome. [file mbio.02159-25-s0007.pdf]

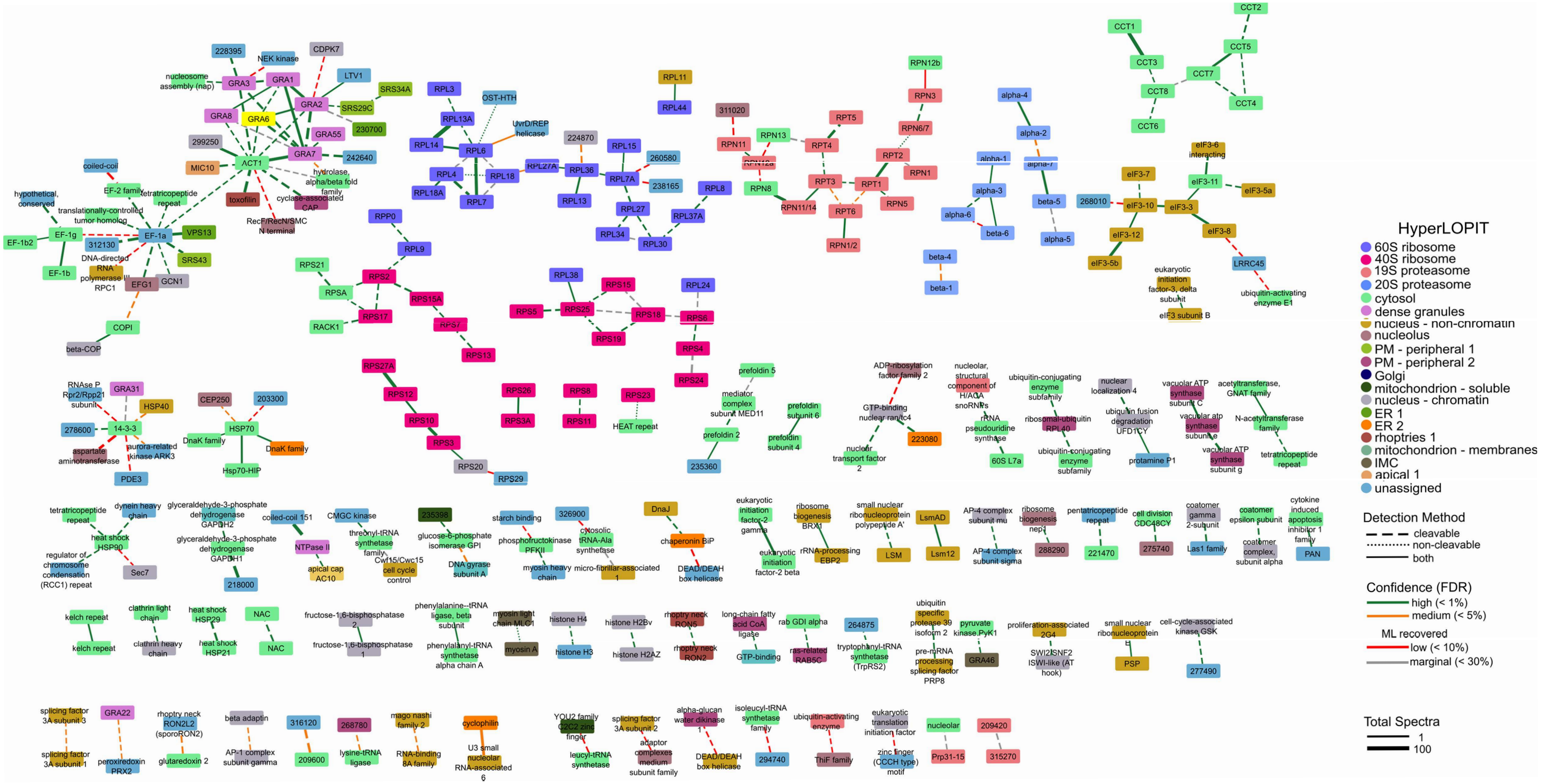

Supplementary Figure S2. T. gondii protein interactome.

The complete interactome of T. gondii cytosolic proteins, integrated with machine learning predictions. Prediction thresholds were stratified based on confidence levels, with high-confidence predictions set at >0.2, medium at >0.5, and low at >0.6. The image is provided in high resolution, ensuring that all node labels are clearly legible.
