# Supplementary material for: Mapping a Toxoplasma gondii interactome by crosslinking mass spectrometry and machine learning
Source: mBio. 2025 Aug 28;16(10):e02159-25. doi: 10.1128/mbio.02159-25 (PMC12505969; doi:10.1128/mbio.02159-25)
Supplement: Legends — for all supplemental files. [file mbio.02159-25-s0008.docx]

**SUPPLEMENTAL DATA LEGENDS:**

**Supplemental Figure S1. SEC Fractions**

The number of crosslinks (CSM) and non-crosslinks (PSM) identified in the each fractions after SEC fractionation. The fraction from 1.18 ml to 1.23 ml had highest ratio (46.6) of corsslink/non-crosslink while the fraction from 1.23 ml to 1.28 ml had highest number (535) of crosslinks. The trace is the UV absorbance at 214 nm representing the amount of peptides.

**Supplementary Figure S2. T. gondii protein interactome.**

The complete interactome of T. gondii cytosolic proteins, integrated with machine learning predictions. Prediction thresholds were stratified based on confidence levels, with high-confidence predictions set at >0.2, medium at >0.5, and low at >0.6. The image is provided in high resolution, ensuring that all node labels are clearly legible.

**Supplemental Movie 1 and 2**

Movies illustrating crosslinks mapped onto cryo-EM structures of the 60S large ribosomal subunit (PDB: 5XXB, Supplemental Movie 1) and the 40S small ribosomal subunit (PDB: 5XXU, Supplemental Movie 2). Interlinks are represented by red lines, whereas intralinks are indicated by blue lines.

**Supplemental File 1A and 1B:**

This file includes the various scripts utilized in this study. Crosslink processing and visualization were conducted using (A) R with the tidyverse package, while machine learning analyses were executed using (B) Python with LightGBM.

**Supplemental File 2**:

Gene ontology (GO) term analysis of the crosslinked proteins was performed, with terms ranked by p-value, revealing a strong representation of typical cytosolic proteins. This analysis utilized ToxoDB version 67, and p-values were calculated using Fisher’s exact test.

**Supplemental File 3:**

Comprehensive PPI table with LightGBM prediction in Microsoft Excel format.

**Supplemental File 4:**

Comprehensive RRI table in Microsoft Excel format.
